# Unusual palmaris longus muscle

**DOI:** 10.4103/0970-0358.41125

**Published:** 2008

**Authors:** P. Thejodhar, Bhagath Kumar Potu, Rakesh G. Vasavi

**Affiliations:** Department of Anatomy, Kasturba Medical College, Manipal University, Manipal - 576 104, Karnataka, India

Dear Sir,

The palmaris longus muscle (PLM) is a slender fusiform muscle, whose short muscle belly arises from the medial epicondyle with a common flexor origin. Its long slender tendon passes palmar to the transverse carpal ligament and is attached to the distal half of its anterior surface and centrally to the Palmar aponeurosis. It is one of the most variable muscles of the human body and is classified as a phylogenetically retrogressive muscle, *i.e*., a short belly with a long tendon.[1] In vertebrates, it is found only in mammals and is best developed where the forelimb is used for ambulation.[2] For example, the Palmaris longus is always present in the orangutan[3] but is variably absent in higher apes such as chimpanzees and gorillas.[2] In humans, the absence of Palmaris longus appears to be hereditary but its genetic transmission is not clear.[3]

During a routine dissection of the forearm of a 39 year-old male cadaver in the Department of Anatomy, Kasturba Medical College (K.M.C), Manipal, we observed that the Palmaris longus muscle on the left side was muscular from its origin right up to the wrist and then continued as the Palmar aponeurosis.

Reimann *et al*, examined 1600 extremities and found incidence rates of 12 and 9% of agenesis and other anomalies respectively. Variations in form constituted 50% of these anomalies. The muscle belly may be central, distal or digastric or it may be completely muscular.[4] Variations also include unilateral absence of the Palmaris longus tendon as well.

Many surgeons agree that the Palmaris longus tendon is the first choice as a donor tendon because it fulfils the necessary requirements of length, diameter and availability, and can be used in reconstructive surgery for a wide variety of procedures including lip augmentation,[5] ptosis correction[6,7] and in the management of facial paralysis[8] without producing any functional deformity.[9]

**Figure 1:**
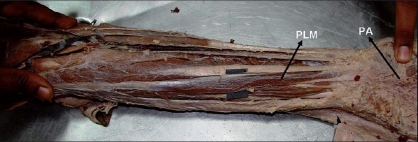
Showing the palmaris longus, which is muscular throughout its length. PLM - palmaris longus muscle, PA - palmar aponeurosis

The Palmaris longus tendon is often considered the ideal donor for tendon grafts for replacement of the long flexors of the fingers and of the Flexor Pollicis longus tendon [Figure 1].[10]

PL anomalies are very important for hand surgeons. In spite of being a landmark to the structures of the wrist, the variations of this tendon may confuse even an experienced surgeon. The clinician must consider this possibility if there is any suspicion of an abnormal swelling in the distal forearm.
